# Fatal cerebral malaria: a venous efflux problem

**DOI:** 10.3389/fcimb.2014.00155

**Published:** 2014-11-06

**Authors:** Ute Frevert, Adéla Nacer

**Affiliations:** ^1^Division of Medical Parasitology, Department of Microbiology, New York University School of MedicineNew York, NY, USA; ^2^Unité de Biologie des Interactions Hôte-Parasite, Département de Parasitologie et Mycologie, Institut PasteurParis, France

**Keywords:** *Plasmodium*, cytoadherence, postcapillary venule, intracranial hypertension, brain edema, CD8+ T cell, macrophage, vascular leakage

## Abstract

Most *Plasmodium falciparum*-infected children with cerebral malaria (CM) die from respiratory arrest, but the underlying pathology is unclear. Here we present a model in which the ultimate cause of death from CM is severe intracranial hypertension. Dynamic imaging of mice infected with *P. berghei* ANKA, an accepted model for experimental CM, revealed that leukocyte adhesion impairs the venous blood flow by reducing the functional lumen of postcapillary venules (PCV). The resulting increase in intracranial pressure (ICP) exacerbates cerebral edema formation, a hallmark of both murine and pediatric CM. We propose that two entirely different pathogenetic mechanisms—cytoadherence of *P. falciparum*-infected erythrocytes in pediatric CM and leukocyte arrest in murine CM—result in the same pathological outcome: a severe increase in ICP leading to brainstem herniation and death from respiratory arrest. The intracranial hypertension (IH) model unifies previous hypotheses, applies to human and experimental CM alike, eliminates the need to explain any selective recognition mechanism *Plasmodium* might use to target multiple sensitive sites in the brain, and explains how an intravascular parasite can cause so much neuronal dysfunction.

## Perspective

Most pediatric cerebral malaria (CM) patients die from respiratory arrest (Waller et al., 1991; Newton et al., 1997), but the underlying pathology is unclear. One suggestion has been that *P. falciparum* causes injury to a sensitive location such as the brainstem, where a small lesion could have fatal consequences (Haldar et al., 2007). Alternatively, death may require a combination of predisposing factors, thus explaining the relatively low mortality rate (WHO, 2011). Here, we discuss insights gained from *P. berghei* ANKA (PbA) infected mice, an accepted model for experimental cerebral malaria (ECM), in the context of recent advances in the understanding of the pathogenesis of human cerebral malaria (HCM). For the purpose of this Perspective, we focus on pediatric HCM, because African children with HCM tend to exhibit more BBB dysfunction, monocyte and platelet accumulation in the brain, and intracranial hypertension (IH) compared to adult HCM patients in Southeast Asia (Hawkes et al., 2013). Pediatric HCM therefore resembles more closely the murine ECM model in terms of clinical features, autopsy findings, and cerebral edema formation.

## Hypotheses for CM pathogenesis

The mechanism by which *P. falciparum* infected red blood cells (iRBC) cause death is not completely understood. Several models have been proposed over the decades to explain the pathogenesis of HCM (Table 1). (1) Extending Frerichs' original *obstruction hypothesis* (Frerichs, 1858), Laveran attributed neurological symptoms and coma to parasites obstructing cerebral capillaries (Laveran, 1884). (2) Marchiafava and Bignami's *sequestration hypothesis* postulates that severe malaria is caused by iRBC accumulating toward the circumference of larger vessels thereby stopping or retarding blood circulation (Marchiafava and Bignami, 1894). In fatal cases of HCM, large numbers of iRBC plug capillaries and reduce their functional lumen (Macpherson et al., 1985). (3) Maegraith's *inflammation hypothesis* proposes that the parasite, whether cytoadherent or not, initiates a chain reaction that allows protein and water to escape through the dysfunctional endothelium, eventually leading to irreversible inflammatory obstruction of the blood flow (Maegraith, 1974). (4) The *cytokine hypothesis* likens HCM to an encephalopathy, in which pathological changes such as edema and coma are of inflammatory origin (Clark and Rockett, 1994). (5) A *combined sequestration and vascular inflammation model* (Grau and De Kossodo, 1994; Hermsen et al., 1997; Postels et al., 2013) holds that endothelial upregulation of adhesion molecules causes iRBC cytoadherence to platelets and leukocyte arrest thereby intensifying endothelial activation and promoting further iRBC sequestration; endothelial death is thought to eventually cause irreversible blood brain barrier (BBB) damage in the absence of vascular occlusion. (6) The *tissue factor model* (Francischetti, 2008) encompasses a series of ordered steps for disease progression with iRBC sequestration as crucial initial event triggering endothelial activation and tissue factor expression thus initiating a cascade of coagulation and inflammation; sequestration of iRBC and platelets then amplifies this cascade resulting in disseminated intravascular coagulation contributing to multi-organ dysfunction and failure. (7) In the PfEMP1 *CIDRα1/EPCR model*, severe pediatric disease is mediated by UpsA *var* genes (DC 8 and 13) expressing iRBC that bind to the endothelial protein C receptor (EPCR) (Moxon et al., 2013; Turner et al., 2013). This interaction inhibits the cytoprotective and anti-inflammatory signaling cascade induced by binding and activation of protein C to EPCR resulting in the activation of protease activated receptor 1 (PAR1) which can trigger a proinflammatory cascade and decrease barrier stability (Xue et al., 2010). Thus, many factors contribute to HCM pathogenesis and this complexity helps explain the apparently contradictory hypotheses proposed over the past century (Van Der Heyde et al., 2006; Idro et al., 2010b). Here, we discuss another model in which the ultimate cause of death from pediatric CM is severe IH. Our model combines prior hypotheses and applies to both ECM and HCM, in particular in pediatric patients with a primarily neurological syndrome (Idro et al., 2010b).

## Lessons from the murine model

Because it is nearly impossible to explore cellular events associated with the collapse of the BBB in HCM patients, we used the PbA-infected CBA/CaJ mouse model to monitor the dynamics of various leukocyte subpopulations within the cortical microvasculature and attempted to correlate our findings with what is known about HCM. In summary, our studies support the hypothesis that ECM-associated pathology is caused by a severe restriction in the venous blood flow due to steric hindrance by arrested leukocytes, not iRBCs, resulting in IH. Using intravital microscopy (IVM), we found more platelets, CD8+ T cells, neutrophils, and macrophages accumulated in postcapillary venules (PCV) from PbA-infected mice with ECM compared to *P. yoelii* XL (PyXL)-infected mice, which develop hyperparasitemia without neurological signs (Nacer et al., 2012, in press). In mice with symptomatic ECM, leukocyte adhesion reduced the functional vascular cross-section of PCV and larger venules significantly and this can explain the reduced cerebral blood flow observed by MRI (Kennan et al., 2005). Supporting our findings, nitric oxide, a key messenger involved in regulation of platelet adhesion and inflammatory and immune responses (Willenborg et al., 2007), decreased both leukocyte accumulation and vascular resistance in venules of PbA-infected mice (Cabrales et al., 2011; Hawkes et al., 2011). Neither capillaries nor arterioles in the cortical microvasculature of mice with ECM nor PCV, arterioles, or capillaries of mice with hyperparasitemia were affected (Nacer et al., in press), thus emphasizing the central role of the postcapillary venule BBB in the ECM-associated vasculopathy (Nacer et al., 2012). Similarly, CD8+ T cells were observed to actively crawl along the wall of larger vessels of the olfactory bulb of PbA-infected mice (Zhao et al., 2014). Because leukocyte adhesion occurred in PCV and larger venules, the center of these vessels remained perfused and complete vascular occlusion was not observed. However, the severe restriction in the venous blood efflux from the brain likely exacerbates edema formation, a hallmark of both ECM and HCM (Sanni, 2001; Penet et al., 2005; Medana and Turner, 2006). Brains from mice with ECM, not hyperparasitemia, have a soft and spongy texture and bulge out of the skull when the dura is accidentally damaged during surgery (Nacer et al., in press). Furthermore, MRI data obtained in mice with ECM provide clear evidence for edema formation, compression of the cerebellum, as well as enlargement and compaction of the brainstem into foramen magnum, all changes compatible with death from IH (Penet et al., 2005).

Arteriolar vasospasms during ECM (Cabrales et al., 2010), due to either increased production of vasoconstrictive mediators or inhibition of vasodilating factors (Martins and Daniel-Ribeiro, 2013) have also been proposed to be important for ECM pathogenesis. Recently, endothelin-1 (ET-1), a vasoactive peptide with inflammatory and platelet-activating properties (Freeman et al., 2014), was shown to be upregulated during both ECM and HCM (Basilico et al., 2002; Dai et al., 2012). C57BL/6 mice displayed neurological signs following the administration of exogenous ET-1 upon infection with PbNK65, a strain that normally does not induce ECM in these mice (Nacer et al., 2012; Martins et al., 2014). Conversely, ECM development was prevented by inhibition of the ET-1 receptor A (Dai et al., 2012). Based on these data, a major role for arteriolar vasoconstriction was proposed for ECM induction in the C57BL/6 mouse model (Freeman et al., 2014). However, ET-1 has a plasma-half life of less than a minute in rodents and it also stimulates the upregulation of endothelial adhesion molecules, promotes leukocyte adhesion, and increases vascular permeability (Callera et al., 2003; King-Vanvlack et al., 2003). Therefore, ET-1 may in fact induce IH (and/or ECM) by restricting the venous blood flow via leukocyte adhesion, preventing adequate brain perfusion via vasoconstriction (Yuh and Dillon, 2010), and inducing BBB disruption. Together, these considerations suggest that a combination of impaired venous efflux and reduced cerebral perfusion causes death from ECM.

## Sequence of events

Cytoadherence of *P. falciparum* expressing a PfEMP1 with a tropism for EPCR increases barrier permeability by interfering with the activation of cytoprotective and anti-inflammatory pathways, eventually culminating in severe childhood malaria (Smith et al., 2013; Turner et al., 2013). Similarly, vascular leakage became apparent 1 day before ECM development, while leukocyte arrest intensified only after the onset of neurological signs (Nacer et al., 2012, in press). Further, PbA-infected mice were shown to exhibit a cytokine storm 24 h prior to the onset of neurological signs with upregulation of a number of cytokines including IL-6, IL-10, and RANTES (Zhao et al., 2014). Thus, it appears that tight junction loosening is independent of iRBC cytoadherence and that vascular leakage precedes leukocyte arrest. In support of this notion, (1) traumatic brain injury is characterized by initial BBB disruption, followed by ICAM-1 upregulation and recruitment of neutrophils, macrophages, platelets, CD8+ and CD4+ T cells (Shlosberg et al., 2010); (2) experimental BBB disruption induces transcriptional changes leading to upregulation of NF-κ B, Stat3, IL-6, CD14, and complement (Cacheaux et al., 2009), events that are frequently associated with severe malaria (Day et al., 1999; Angulo and Fresno, 2002; Tripathi et al., 2009; Higgins et al., 2011; Liu et al., 2012; Noone et al., 2013); and (3) TNF-α, a cytokine with barrier permeability increasing capabilities (Tsao et al., 2001) that has also been linked to BBB disruption in various hepatic encephalopathies (Cui et al., 2013), is involved in the pathogenesis of both ECM and HCM (Hunt et al., 2006) together with several other members of the TNF superfamily (Randall et al., 2010). Together, these findings suggest that leukocyte accumulation coincides with the final phase of CM.

## Intracranial hypertension in HCM

Over two decades ago, Newton and colleagues associated increased intracranial pressure (ICP) with poor outcomes in pediatric HCM patients in Kenya (Newton et al., 1991). Severe IH is difficult to diagnose based on clinical signs. The most reliable clinical indicator for ICP spikes has been pupil dilation with sluggish or absent responses to light (Newton et al., 1997). Using an invasive monitor to measure the opening pressure during lumbar puncture, two studies in Kenyan and Gambian children with HCM documented a correlation between increased ICP and poor prognosis, i.e., coma and death, or severe neurological sequelae in survivors (Waller et al., 1991). A more recent Kenyan study confirmed the close correlation between HCM and increased ICP in pediatric HCM, but also showed that repeated lumbar opening pressure measurements are required to reveal the maximum ICP or the temporal relationship between ICP and seizures or agonal events (Beare et al., 2012).

Transcranial Doppler is now increasingly being used as an important non-invasive tool in neurocritical care to estimate ICP and cerebral perfusion pressure (Saqqur et al., 2007). IH forces the CSF into the perineural space between the dura mater and the optic nerve causing the optic nerve sheath (ONS) to swell. ONS diameters can be measured by ultrasonography and increases in diameter correlate with IH (Le et al., 2009). Ultrasonography is feasible even in low-resource settings where most pediatric HCM patients are treated. In Malawi, bedside hand-held devices revealed increased ONS diameters in all children diagnosed with HCM (Murphy et al., 2011). In Uganda, neurological sequelae were significantly more common in children with increased ONS diameter, although fatalities were similar in children with and without increased ONS diameter (Murphy et al., 2011; Beare et al., 2012). These findings prompt questions. Are vascular alterations observed by retinoscopy such a good predictor of fatal HCM, because ONS swelling impairs perfusion of the retinal vein at the optic canal—similar to compression of the brainstem? How reversible are vascular injuries in the retina if the child survives and do they occur in patients who never enter the agonal phase of the disease?

IH-mediated compression of respiration centers in medulla and pons can explain why many children die from respiratory arrest despite adequate pulse and blood pressure (Waller et al., 1991; Newton et al., 1997). Indeed, comparison of MRI and autopsy data from pediatric patients recently identified brainstem herniation due to increased brain volume as the strongest predictor of death HCM (Seydel et al., 2011; Kampondeni et al., 2013). In addition, mechanical vascular injury due to stretching during caudal displacement of the brainstem through the foramen magnum explains the marked hemorrhaging associated with HCM (Yuh and Dillon, 2010). Together, these data suggest a direct link between IH and fatal HCM.

## Neurological symptoms and sequelae

Differences in duration and intensity of IH can explain the variability in severity of neurological sequelae in HCM survivors (Idro et al., 2010b). IH, repeated and prolonged seizures, as well as deep and prolonged coma are known risk factors for poor outcome predominantly in pediatric HCM (Van Hensbroek et al., 1997; Idro et al., 2006), but how these factors lead to IH and neural injury is unknown (Idro et al., 2010a; Beare et al., 2012). Global compression and hypoperfusion of the brain due to edema and general swelling offers an explanation for the cognitive deficits, behavioral difficulties and neuropsychiatric sequelae suffered by many HCM survivors. Analogous to brainstem herniation through the foramen magnum and its effect on respiration (Kampondeni et al., 2013), herniation into other foramina of the skull could compress the olfactory, optic, vestibulocochlear, glossopharyngeal, and hypoglosseal nerves as well as the accompanying blood vessels. Resulting damage can explain, at least in part, other major long-term sequelae such as deficits in vision, hearing, and smell. The notion that IH can be caused by sequestration of iRBC as well as leukocytes is supported by reports documenting that both HCM and ECM are associated with deafness, loss of smell, and blindness (Brewster et al., 1990; Schmutzhard et al., 2010, 2011; Idro et al., 2010a; Saggu et al., 2011; Zhao and Mackenzie, 2011; Zhao et al., 2014). Thus, IH eliminates the need to explain any selective recognition mechanism *Plasmodium* might use to target multiple sensitive sites in the brain (Idro et al., 2010b). Further, leukocyte sequestration likely also plays a role in IH during *P. falciparum* HCM, because artesunate treatment did not rescue patients with a low sequestered parasite biomass (White et al., 2013) and also had a higher efficacy in predominantly adult Asian patients compared to African children, who exhibit significantly more mononuclear cell sequestration (Silamut et al., 1999; Dorovini-Zis et al., 2011), particularly the CM2 group (Milner et al., 2013). Thus, human and murine CM induce very similar neurological symptoms and signs, respectively, including non-life-threatening neurological impairments.

The CM-associated pathology is similar to that found in other IH-driven neurological disorders. In idiopathic IH, for example, impaired venous outflow from the brain can cause headache, nausea, vomiting, and altered consciousness, including stupor or coma in severe cases (Yuh and Dillon, 2010). Other symptoms include pulsatile tinnitus and visual alterations up to blindness due to swelling of the optic disc. As for HCM, the essential diagnostic criterion is an increased lumbar puncture opening pressure (Yuh and Dillon, 2010) and the central retinal vein pressure is used as surrogate for ICP measurement (Harder et al., 2007; Jonas et al., 2008). Occasionally, idiopathic IH can lead to tonsillar herniation, brainstem compression, and death (Yuh and Dillon, 2010).

## The intracranial hypertension model

**Figure 1:**
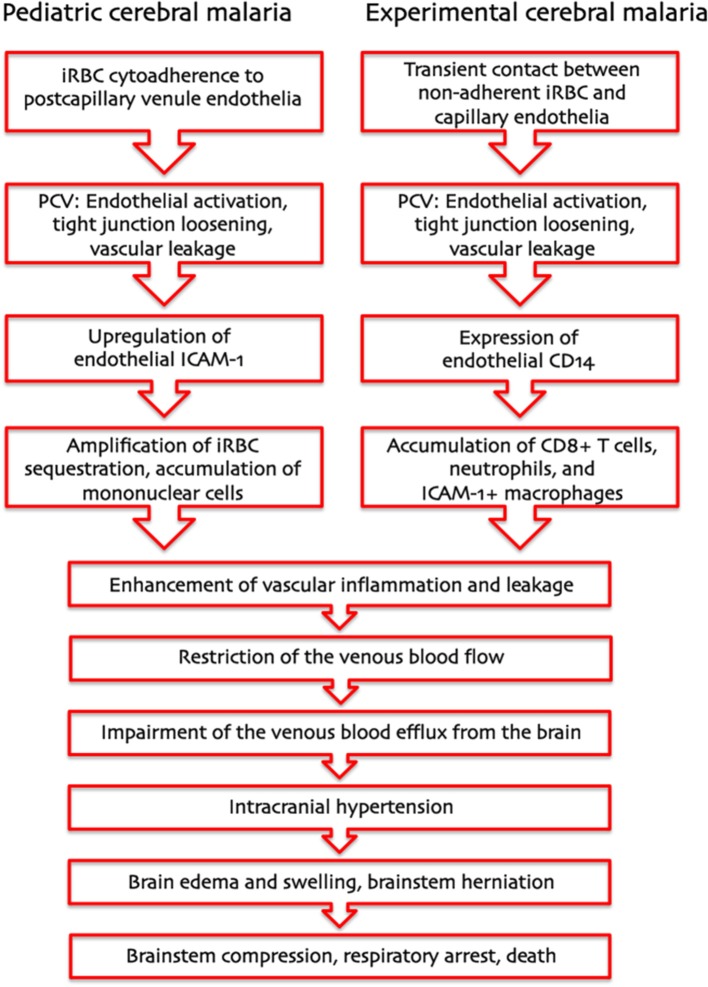
**Pathogenesis of human and murine cerebral malaria—proposed sequence of events**. The intracranial hypertension hypothesis explains how two entirely different pathogenetic mechanisms—cytoadherence of *P. falciparum*-infected erythrocytes in pediatric CM and leukocyte arrest in murine CM—can result in the same pathological outcome: a severe increase in intracranial pressure leading to brainstem herniation and death from respiratory arrest.

Based on these considerations, we propose that two entirely different pathogenetic mechanisms—cytoadherence of *P. falciparum* iRBC in pediatric HCM and leukocyte arrest in ECM—drive a severe restriction in the venous blood flow that results in the same pathophysiological outcome: IH (Figure 1). In HCM, the primary function of sequestration of *P. falciparum* iRBC is considered to be an immune evasion mechanism allowing parasites to avoid splenic clearance (Safeukui et al., 2008; Buffet et al., 2009). *P. falciparum* iRBC sequestration is known to occur in all organs, not only the brain. Therefore, upregulation of adhesion molecules, at any site, would increase sequestration and disrupt the venous blood flow. However, enclosure into the skull allows little room for expansion, rendering the brain especially prone to serious damage. Regarding ECM, PbA infection causes the recruitment of larger numbers of activated leukocytes compared to infection with PyXL (Nacer et al., 2012, in press). Leukocyte accumulation, like iRBC sequestration, reduces the venous efflux and raises the ICP, in severe cases potentially leading to brainstem compression and respiratory arrest. The IH model unifies previous hypotheses, applies to the pathogenesis of HCM and ECM alike, and explains how a largely intravascular parasite can cause so much neuronal dysfunction, even in the absence of cytoadherence, and why anti-malarial treatment can reverse coma so rapidly and without significant tissue necrosis despite large numbers of iRBC in the brain of most patients. Further, variations in severity and/or duration of IH can explain why some children have a poor neurological outcome, while others improve rapidly with minimal deficits despite similar clinical presentation.

Thus, despite fundamental differences in parasite biology, the PbA-infected mouse model is suitable for study of blood flow alterations in the human brain that are not readily accessible in *P. falciparum*-infected individuals. Perhaps more relevant, the leukocyte arrest-induced venous blood flow impairment can also explain the increasingly reported severe complications in monoinfections in *P. vivax*, an essentially non-cytoadherent *Plasmodium* species, which tend to take a similar course as *P. falciparum* infections (Beg et al., 2002; Sharma et al., 2012; Sarkar et al., 2013; Goyal and Makwana, 2014). The shared predilection for reticulocyte invasion (McNally et al., 1992; Cromer et al., 2006; Panichakul et al., 2007; Russell et al., 2011), the marked proinflammatory responses, and the reversible microvascular dysfunction typically associated with *P. vivax* infections (Thapa et al., 2007; Andrade et al., 2010) all suggest that the PbA-infected mouse model is also suitable for study of the pathogenesis of severe *P. vivax* malaria, including HCM. The PbA-mouse ECM model can reveal reversible agonal events obscured in autopsy material and allows *in vivo* monitoring techniques such as IVM on the microscopic and MRI on the macroscopic level that can help identify the ultimate cause of death from HCM. Clinical efforts to maintain normal ICP and cerebral perfusion and to avoid factors that exacerbate IH, as recommended for treatment of idiopathic IH, may also help patients survive the critical phase of HCM with minimal neurological sequelae. When resources allow, perhaps decompressive craniotomy, a successful strategy in idiopathic IH patients, could save the lives of children living in *P. falciparum* endemic areas.

## Conclusions and future directions

In agreement with Marchiafava's and Bignami's hypothesis that iRBC sequestration retards the blood flow in larger veins during the agonal period of severe malaria (Marchiafava and Bignami, 1894), we argue that venous blood flow impairment is sufficient to increase the ICP to a point that the resulting cerebral edema leads to brainstem herniation and death from respiratory arrest. Simultaneous venous efflux restriction and continued influx of arterial blood necessarily exacerbates vascular injury and promotes hemorrhaging. Because the majority of successfully treated comatose HCM patients recover without permanent neurological deficits (Brewster et al., 1990; White and Ho, 1992; Idro et al., 2010b), many of the pathological alterations detected in autopsy specimens may not adequately reflect the cerebral blood perfusion before death. Further, iRBC sequestration in larger vessels allows continued perfusion so that distinction between capillaries and PCV is crucial to evaluate blood flow obstruction prior to the agonal phase of the disease. Because size and morphology suggest that most of the published blood vessels with cytoadherent *P. falciparum* iRBC are PCV and because the blood flow in murine PCV continues despite leukocyte arrest, we propose that the contribution of vascular occlusion to HCM pathogenesis is less important than frequently assumed. Longitudinal ICP measurements, MRI-based monitoring of the brain, IVM, and—once available—high-resolution imaging of the retinal microvasculature, in patients or murine models, are expected to reveal the ultimate cause of death from CM.

## Of mice and men

We hope to have conveyed to the reader that there is value to the ECM PbA model even without it strictly replicating observations in human disease caused by *P. falciparum*. For example, in our view, *P. berghei* has not been conclusively shown to cytoadhere *in vivo* or sequester within infected mice. Our contention is that another mechanism, leukocyte adhesion, produces the same end point. Sadly, in order to make this model acceptable, parallels with *P. falciparum* HCM are sought where none exist. Even more so, because the focus on the unique aspects of *P. falciparum* infection, cytoadhesion and sequestration, may distract from pathogenic mechanisms that may be equally important, for example for complicated *P. vivax* infections. Many labs have attempted to demonstrate *P. vivax* iRBC cytoadhesion in order to justify the occurrence of clinical CM in patients infected with this parasite. This is tragic because the presence of all parasite stages in the blood suggests that *P. vivax* is essentially non-cytoadherent. Thus, iRBC sequestration is no longer sufficient to explain the pathogenesis of CM.

### Conflict of interest statement

The Review Editor Samuel Wassmer declares that, despite being affiliated to the same institution as author(s) Ute Frevert, the review process was handled objectively. The authors declare that the research was conducted in the absence of any commercial or financial relationships that could be construed as a potential conflict of interest.
