# Supplementary material for: Fatal cerebral malaria: a venous efflux problem
Source: Front Cell Infect Microbiol. 2014 Nov 6;4:155. doi: 10.3389/fcimb.2014.00155 (PMC4222339; doi:10.3389/fcimb.2014.00155)
Supplement: Supplementary file 1 [file Table1.PDF]

Table 1. Hypotheses for the pathogenesis of cerebral malaria.

Hypothesis	Proposed mechanism of pathogenesis	References
Obstruction	Parasite blockage of cerebral capillaries	Frerichs, 1858; Laveran, 1884
Sequestration	Accumulation of iRBC at the periphery of larger vessels leading to a reduction or blockage in circulation. Reduction in functional lumen and obstruction of capillaries	Marchiafava and Bignami, 1894; MacPherson et al, 1985
Inflammation	Presence of the iRBC, independent of cytoadherence, leads to endothelial dysfunction resulting vascular leakage. Blood flow obstruction then promotes irreversible inflammatory stasis	Maegraith, 1974
Cytokines	Malarial encephalopathy caused by inflammatory responses	Clark and Rockett, 1994
Combined sequestration and vascular inflammation	Endothelial activation with concurrent upregulation of adhesion molecules leads to iRBC cytoadherence to platelets resulting in further endothelial activation and parasite adhesion. Endpoint is irreversible BBB damage due to endothelial cell death, independent of vascular obstruction	Grau and de Kossodo, 1994; Hermsen et al 1997; Postels et al, 2013
Tissue factor	iRBC sequestration initiates a cascade that triggers endothelial activation, tissue factor expression, coagulation and inflammation. Sequestration of platelets and iRBC intensifies widespread intravascular coagulation ultimately leading multi-organ failure	Francischetti, 2008
CIDRα1/EPCR	Parasites expressing UpsA *var* genes bind to EPCR. Inhibition of cytoprotective and anti-inflammatory signaling cascade triggered by the binding of APC to EPCR. Alternate pro-inflammatory pathway initiated by activation of PAR1 leading to decreased barrier stability	Moxon et al, 2013;Turner et al, 2013
Intracranial hypertension	Presence of the parasite initiates a cascade of events that begins with barrier disruption leading to the recruitment of leukocytes followed by neurological signs. Accumulation of iRBC causes severe reductions in the functional lumen of post-capillary venules. Resulting reductions in venous efflux increase ICP, the resulting intracranial hypertension can lead to brainstem compression and respiratory arrest	Newton et al, 1991; Waller et al, 1991; Nacer et al, 2014

iRBC, infected red blood cell; EPCR, endothelial protein C receptor; APC, activated protein C; PAR1, protease activated receptor 1; ICP, intracranial pressure.
